# Supplementary material for: Phenotypic Diversity and Genetic Parameters of Coffea canephora Clones
Source: Plants (Basel). 2023 Dec 1;12(23):4052. doi: 10.3390/plants12234052 (PMC10707845; doi:10.3390/plants12234052)
Supplement: Supplementary file 1 [file plants-12-04052-s001.zip › plants-2698149-supplementary.pdf]

plants

MDPI

Supporting Information

Phenotypic Diversity and Genetic Parameters of *Coffea canephora* Clones

Caroline de Souza Bezerra 1, Jennifer Souza Tomaz[1], Mágno Sávio Ferreira Valente2, Marcelo Curitiba Espíndula3, Ricardo Lívio Santos Marques[1], Hugo César Tadeu[1], Fábio Medeiros Ferreira[1], Rogério Freire da Silva4, Carlos Henrique Salvino Gadêlha Meneses4 and Maria Teresa Gomes Lopes[1],*.

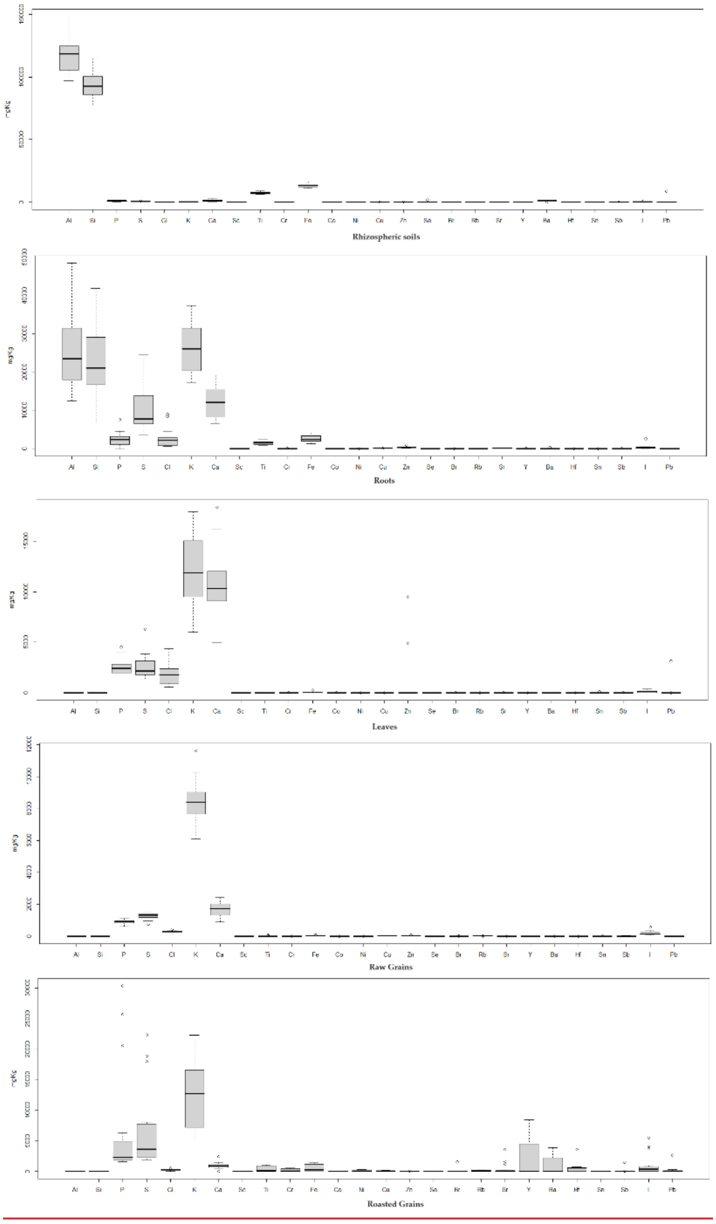

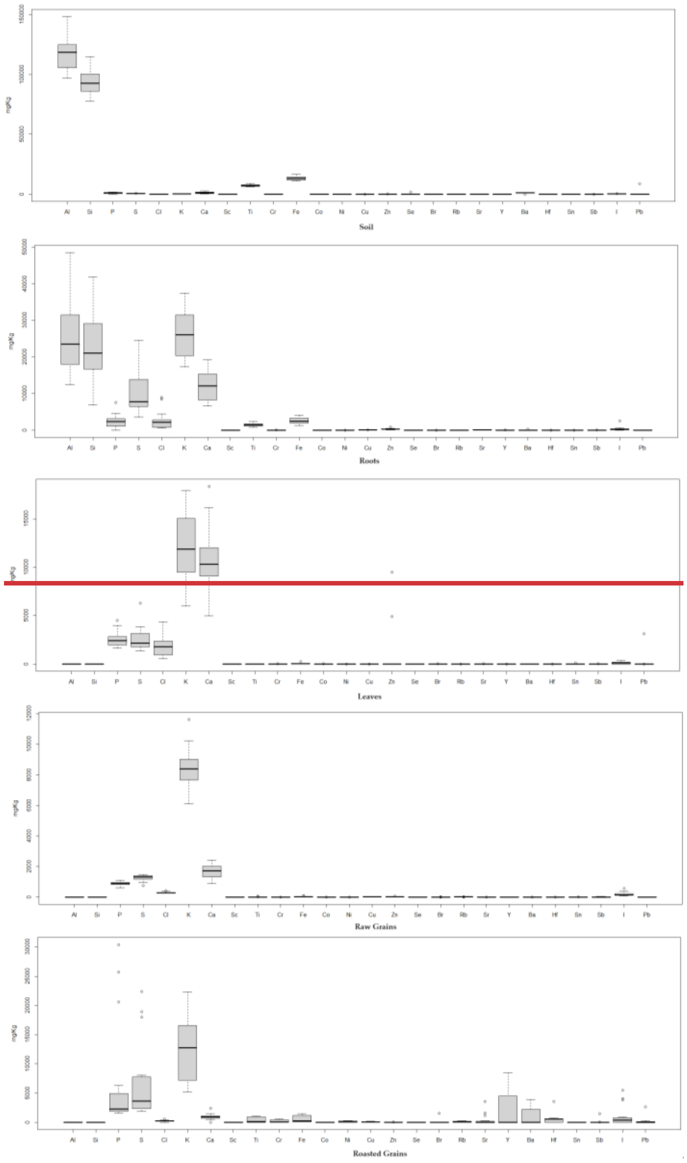

Figure S1. Comparison of the inorganic components analyzed in rhizospheric soils ~~soil~~, root, leaves, raw and roasted beans of *C. canephora.* Boxes show the 25th and 75% quartiles, bars show the 10th and 90% quartiles, filled squares show the median, empty circles show outliers. Significant at 5% by ANOVA.

plants

MDPI

Table S1. Values of average concentrations of chemical elements of leaves (mg.kg-1), productivity, and grain yield (60 kg bags.ha-1) separated by the Scott-Knott test and standard deviation of 15 clones of *C. canephora*, evaluated in Manaus-AM.

Clones	Phosphorus	Sulfur	Chlorine	Potassium	Calcium	Titanium	Vanadium	Chromium	Iron	Cobalt	Nickel	Copper
BRS3137	4,064.25 a	3,979.75 a	4,590.50 a	2,7275.00 a	14,884.75 a	8.65 a	0.22 a	2.06a	110.97 a	0.36 b	0.98 a	29.05 b
	± 1,810.68	± 1,264.11	± 4,915.83	14,565.64	± 5,173.59	± 4.01	± 0.45	± 2.32	± 25.32	± 0.11	± 0.56	± 24.05
BRS3213	4,955.25 a	2,195.25 a	3,236.50 a	2,5097.50 a	10,068.00 a	7.75 a	0.26 a	0.75 a	65.01 a	0.25 b	0.61 a	19.12 b
	± 2,736.47	± 229.96	± 1,947.35	1 6,118.71	± 7,619.14	± 3.56	± 0.31	± 0.34	± 23.04	± 0.04	± 0.37	± 15.39
BRS1216	4,251.75 a	3,486.00 a	2,861.75 a	29,609.75 a	9,551.25 a	2,021.11 a	0.17 a	272.84 a	74.87 a	0.16b	697.96 a	27.22 b
	± 3,611.73	± 1,524.05	± 1,892.19	± 2,7676.40	± 3,629.64	± 4,027.26	± 0.35	± 544.11	± 25.06	± 0.05	± 828.19	± 26.74
BRS2314	2,826.00 a	2,266.00 a	1,903.45 a	17,331.50 a	9,695.25 a	1,211.05 a	0.23 a	4.15 a	113.08 a	12. 07 a	276.83 a	16.91 b
	± 839.47	± 388.54	±824.74	± 6,488.47	± 4,744.60	± 2,409.30	± 0.27	± 7.02	± 87.16	± 9.64	± 551.45	± 13.76
BRS3210	3,958.00 a	5,114.25 a	3,235.17 a	24,170. 00 a	19,200.75 a	908.69 a	0.43 a	0.67 a	115.02 a	0.53 b	0.33 a	25.37 b
	± 3,725.08	± 6,274.72	± 4,377.94	± 17,037.83	± 28,408.39	± 1,795.56	± 0.86	± 0.20	± 125.69	± 0.45	± 0.16	± 18.86
BRS3220	3,057.50 a	3,248.50 a	2,542.05 a	17,892.50 a	14,530.25 a	1,534.48 a	0.17 a	3.46 a	115.57 a	0.63 b	619.12 a	3,073.05 a
	± 1,350.97	± 2,271.22	± 1,514.70	± 7,051.88	± 14,149.59	± 1,761.32	± 0.35	± 5.67	± 152.18	± 0.22	± 1,237.25	± 3,427.56
BRS2336	2,300.25 a	2,152.25 a	1,853.50 a	11,816.50 a	11,388.25 a	1,998.58 a	0.26 a	0.58 a	66.94 a	0.25 b	0.6 a	16.94 b
	± 187.35	± 784.96	± 764.75	± 1,886.43	± 5,772.34	± 2,352.51	± 0.17	± 0.22	± 27.01	± 0.01	± 0.21	± 12.64
BRS2299	2,206.25 a	2,448.50 a	1,906.27 a	13,414.25 a	12,910.75 a	3,824.84 a	0.00 a	0.53 a	78.11 a	0.26 b	0.26 a	11.22 b
	± 723.27	± 643.47	± 1,522.72	± 6,185.19	± 4,435.85	± 3,289.63	± 0.01	± 0.14	± 41.57	± 0.24	± 0.07	± 4.08
BRS3193	1,511.75 a	1,926.50 a	2,220.55 a	9,273.75 a	13,490.00 a	3,179.96 a	0.19 a	0.47 a	94.05 a	0.32 b	0.11 a	22.18 b
	± 273.14	± 586.28	± 1,429.27	± 3,540.44	± 3,392.41	± 4,128.86	± 0.26	± 0.10	± 55.58	± 0.18	± 0.02	± 16.56
Clone 12	2,866.00 a	2,459.75 a	2,078.22 a	16,155.00 a	12,797.00 a	2,090.25 a	0.00 a	291.33 a	112.30 a	0.35 b	0.45 a	32.93 b
	± 997.44	± 577.33	± 1,053.25	± 6,693.41	± 3,371.48	± 4,152.51	± 0.01	± 581.11	± 33.72	± 0.12	± 0.18	± 28.79
BRS2357	2,065.75 a	2,085.00 a	1,123.37 a	13,459.00 a	10,187.50 a	2,449. 79 a	0.14 a	0.68 a	65.81 a	0.24 b	0.44 a	16.55 b
	± 738.81	± 507.14	± 612.12	± 5,786.39	± 2,642.31	± 3,166.04	± 0.29	± 0.15	± 14.01	± 0.10	± 0.22	± 6.49
RO_C125	2,578.25 a	2,552.75 a	1,655.57 a	14,405.50 a	18,992.50 a	2,275.82 a	0.31 a	0.52 a	72.12 a	0.44 b	0.44 a	23.75 b
	± 1,092.72	± 1,034.73	± 1,338.32	± 6,256.34	± 15,010.51	± 4,541.45	± 0.40	± 0.27	± 56.12	± 0.17	± 0.27	± 15.07
RO_C160	2,285,00 a	2,730. 00 a	1,216.45 a	13,378.50 a	13,385.50 a	4,234.20a	0.29 a	316.29 a	57.13 a	0.15 b	270.09 a	14.33 b
	± 836.36	± 1,064.12	± 366.00	± 5,310.83	± 6,679.05	± 2,954.31	± 0.20	± 630.47	± 32.18	± 0.04	± 538.61	± 5.66
Clone 9	2,254.00 a	3,075.75 a	1,477.75 a	12,895.00 a	9,173.75 a	2,128.21 a	0.06 a	2.09 a	71.12 a	0.16 b	372.86 a	16.73 b
	± 1,174.95	± 2,125.43	± 691.06	± 4,182.7	± 2,967.19	± 2,486.80	± 0.13	± 3.25	± 30.71	± 0.01	± 742.76	± 8.00

Clone 15	2,988.00 a	2,652. 00 a	1,661.75 a	15,602.50 a	10,543.50 a	2,067.53 a	0.06 a	277.14 a	57.27 a	0.13 b	276.75 a	1,966.91 a
	± 1,091.17	± 779.76	± 443.62	± 9,823.72	± 3,043.61	± 2,426.24	± 0.12	± 549.24	± 7.47	± 0.04	± 550.83	± 3,903.4

Means followed by the same letters constitute a statistically homogeneous group, at 5% probability.

Continuation…

Clones	Zinc	Bromine	Rubidium	Strontium	Yttrium	Hafnium	Iodine	Grain yield	Productivity
BRS3137	17.49 a	12.13 a	22.40 b	28.77 a	1,032.84 a	3.82 b	719.10 a	0,285 c	29,32 c
	± 8.18	± 18.05	± 17.81	± 9.90	± 2,057.44	± 1.34	± 331.42	± 0.01	± 5.38
BRS3213	18.10 a	20.75 a	23.68 b	17.04 a	10.045 a	3.75 b	47.10 c	0,200 c	30,77 c
	± 2.94	± 10.46	± 27.59	± 9.99	± 9.22	± 1.88	± 60.13	± 0.04	± 7.26
BRS1216	26.73 a	16.14 a	25.06 b	14.76 a	1,505.33 a	1,187.72 b	198.61 c	0,288 a	116,53 a
	± 14.89	± 17.17	± 25.54	± 4.30	± 3,003.11	± 1,368.94	± 369.66	± 0.03	± 25.94
BRS2314	14.85 a	9.01 a	2,294.11 b	17.33 a	811.67 a	1,081.73 b	48.92 c	0,303 b	75,32 b
	± 13.26	± 1.64	± 4,569.93	± 8.93	± 1,616.88	± 2,154.84	± 63.51	± 0.08	± 20.00
BRS3210	2,208.56 a	865.75 a	25.41 b	52.03 a	1,703.43 a	1,373.30 b	30.56 c	0,253 c	32,04 c
	± 4,356.38	± 1713.50	± 12.82	± 63.84	± 2,119.44	± 1,604.54	± 54.14	± 0.03	± 5.24
BRS3220	3,432.96 a	268.16 a	6,438.32 a	1,818.81 a	2,078.39 a	4,666.50 a	312.55 b	0,228 c	42,83 c
	± 3,951.51	± 517.90	± 4,377.01	± 3,573.5	± 1,472.11	± 2,232.85	± 429.68	± 0.01	± 17.68
BRS2336	12.46 a	462.93 a	5,534.25 a	22.49 a	2,713.50 a	3,959.25 a	356.86 b	0,243 b	69,91 b
	± 4.33	± 914.05	± 1,126.29	± 10.71	± 940.06	± 1,708.77	± 56.15	± 0.02	± 10.38
BRS2299	1,404.15 a	2,347.67 a	6,102.00 a	21.33 a	2,221.50 a	1,406.07 b	241.37 b	0,278 b	89,74 b
	± 2,787.24	± 4,654.22	± 750.97	± 7.23	± 505.89	± 1,625.89	± 131.62	± 0.05	± 37.52
BRS3193	12.34 a	691.44 a	2,958.33 b	25.52 a	3,804.00 a	2,701.50 a	41.95 c	0,253 c	48,43 c
	± 5.85	± 803.10	± 2,364.99	± 6.53	± 2,822.77	± 191.82	± 83.9	± 0.02	± 8.85
Clone 12	2,404.52 a	3,016.93 a	1,036.14 b	21.50 a	2,616.27 a	1,841.19 b	24.39 c	0,208 c	31,98 c
	± 4,725.67	± 4,399.66	± 2,045.92	± 5.07	± 1,754.71	± 1,281.70	± 48.78	± 0.08	± 21.44
BRS2357	2,256.34 a	3.97 a	5,161.86 a	18.17 a	1,920.03 a	3,068.22 a	0.00 c	0,300 a	102,12 a
	± 4,490.04	± 4.70	± 4,240.70	± 6.36	± 1,314.35	± 2,297.74	± 0.01	± 0.01	± 14.68
RO_C125	2,111.93 a	2,673.83 a	5,984.23 a	1,823.67 a	2,877.50 a	4,881.25 a	45.65 c	0,293 c	64,25 c
	± 4,190.72	± 2,335.06	± 4,257.74	± 3,546.92	± 412.83	± 2,302.35	± 91.30	± 0.01	± 11.77
RO_C160	2,092.92 a	1,647.95 a	4,673.47 a	2,123.82 a	2,829.52 a	3,061.25 a	0.00 c	0,300 a	111,71 a
	± 4,159.39	± 1,106.89	± 4,650.24	± 4,188.79	± 2,078.93	± 513.77	± 0.01	± 0.03	± 24.88
Clone 9	2,158.18 a	1,989.38 a	2,870.06 b	17.86 a	1,377.98 a	2,767.00 a	349.13 b	0,270 a	99,53 a

	± 4,285.89	± 2,172.74	± 2,427.52	± 1.05	± 1,620.48	± 618.39	303.30	± 0.02	± 7.99
Clone 15	3,187.71 a	2,032.90 a	428.18 b	18.98 a	3,014.25 a	2,826.27 a	0.00 c	0,263 b	81,45 b
	± 3,856.10	± 2,937.97	± 828.55	± 4.74	± 735.54	± 1,977.89	± 0.01	± 0.03	± 14.38

Means followed by the same letters constitute a statistically homogeneous group, at 5% probability.
